# Sanatorium Figures

**Published:** 1920-04-24

**Authors:** 


### Sanatorium Figures.

### Under Ideal and Makeshift Conditions.

Attention is drawn to a recent report of the Research Committee on Mortality after Sanatorium Treatment of Phthisis, for which Dr. Noel Bardswell is mainly responsible. It is of particular interest just at this time when the whole question of sanatorium treatment and after-treatment is under discussion, and when an entirely new process has been foreshadowed. Local authorities will doubtless have to provide considerably more accommodation. The medical correspondent of the *Manchester Guardian* discusses Dr. Bardswell's analysis of the circumstances at the King Edward VII. Sanatorium at Midhurst, which he selected for his investigations. This sanatorium was established on a carefully selected site; it was built according to the latest doctrines of the time, lavishly equipped, and staffed with the ablest of physicians and nurses. It seemed like an ideal condition for a curative sanatorium. The report states that the average age of Midhurst patients, drawn from the professional classes, was about thirty, yet the rates of mortality experienced by those discharged, with the disease arrested or much improved, were those shown by the general population.

### Significant Deductions.

These figures (the correspondent points out) are important for any local authorities preparing schemes for the extension of hospital or sanatorium accommodation, not in order to discourage them in making provision, but in order that their efforts may be directed to the true end of prevention. The establishment of a sanatorium in the country, the acquisition of large tracts of land at great cost, and so forth, by local authorities could not hope to compete with Midhurst, yet the Midhurst figures show that the results obtained there in medium and advanced cases are little, if any, better than can be claimed for local hospitals that are avowedly makeshifts. It may be long before we can obtain early diagnosis and treatment, except in the case of school-children. Wage-earners, fears the correspondent, will continue to work until their condition becomes obvious and incurable. Sanatorium treatment even then is not useless; it will add to their comfort and perhaps prolong their lives, and, above all, it will provide them with training in personal hygiene, and at least reduce the risk of infection to friends and relatives. It is therefore advocated that the aim should be the provision of a maximum number of beds with the money available, and that the authorities should disabuse their minds of the idea of providing about an acre of land per patient, or removing the sufferers a distance from their homes, in the illusory quest of some climate advantage. The object should be to teach the patient how the essentials of sanatorium conditions and discipline may be reproduced to some extent in the humblest dwelling. In this way such treatment may be made a really useful adjunct to all the other preventive measures which alone will rid us of the "crime" of tuberculosis.

Village settlements, however, come within a different category, for they aim at making permanent provision, where the question of mortality after treatment hardly arises at all, as treatment is more or less permanent. A deputation recently waited on the Ministry of Health to urge the establishment of such settlements—mainly concerning ex-Service men—and the case put before the Minister was, broadly, that sanatorium treatment for tuberculosis, even where accompanied by training in a suitable occupation, has been found to be inadequate. The general experience has been that patients who return from a sanatorium to their homes and former occupations are unable permanently to earn a living or maintain their health. The interest both of the patients and of the community requires that patients should pass through a threefold course, first of sanatorium treatment, secondly of training, and thirdly of permanent settlement in suitable surroundings. The village settlement should be a natural development of the sanatorium and training colony, and the patient should be in a position to look forward to being able, on completion of his course of treatment and training, to take up his permanent residence in a settlement where, still in close touch with the sanatorium, he could work under conditions which would enable him to maintain his health, and have his family or dependants with him. In the settlement the patient and his family would have to be housed, the necessary workshops and other buildings would have to be provided, and (in the case of the civilian) the patient's earnings would have to be supplemented; but the community would gain in the result by the prevention of the spread of infection, and the fact that the tuberculosis patient would remain a productive worker. As illustrating the results of establishing a village settlement on these lines, it was stated that at the Cambridgeshire Tuberculosis Colony, out of thirty cases who have passed from sanatorium treatment and training into the settlement there, not one has died in four years.

### Minister's Goodwill.

On the question of village settlements, Dr. Addison assured the deputation of his good-will. There are, however, various difficulties to be overcome. The success of a village settlement depends even more upon the personality of the man in charge than upon the material provision made. As regards finance, the provision of ten settlements for not less than 200 patients each, as proposed by the Interdepartmental Committee, will, he considers, cost much more than the sum of £1,000,000 suggested by the Committee. Again, as Minister of Health, he cannot consider only the case of the ex-soldier; he has to consider also the civilian population, who have no pensions to supplement their earnings. Further, the Cambridgeshire Tuberculosis Colony is managed by a voluntary organisation; but in the establishment of further settlements it will be necessary to rely, in the main, upon local authorities.

The problem is therefore one of much complexity; but a comprehensive scheme, dealing with all the various issues involved, is under discussion with the Treasury. There is, in addition, the important question of the situation of the projected settlements. On this, Dr. Addison intimated that Dr. Nathan Raw and Dr. Varrier Jones have consented to undertake, in conjunction with a Medical Officer of the Ministry, a series of local investigations with a view to advising him on this matter.

